# Event-Related Potential Markers of Suicidality in Adolescents

**DOI:** 10.1093/ijnp/pyad039

**Published:** 2023-07-09

**Authors:** Deniz Doruk Camsari, Charles P Lewis, Ayse Irem Sonmez, Can Ozger, Parmis Fatih, Deniz Yuruk, Julia Shekunov, Jennifer L Vande Voort, Paul E Croarkin

**Affiliations:** Department of Psychiatry and Psychology, Mayo Clinic, Rochester, Minnesota, USA; Department of Psychiatry and Psychology, Mayo Clinic, Rochester, Minnesota, USA; Department of Psychiatry and Behavioral Sciences, University of Minnesota, Minneapolis, Minnesota, USA; Department of Psychiatry and Psychology, Mayo Clinic, Rochester, Minnesota, USA; Department of Psychiatry and Behavioral Sciences, University of Minnesota, Minneapolis, Minnesota, USA; Department of Psychiatry and Psychology, Mayo Clinic, Rochester, Minnesota, USA; Department of Psychiatry and Psychology, Mayo Clinic, Rochester, Minnesota, USA; Department of Psychiatry, Rush University, Chicago, Illinois, USA; Department of Psychiatry and Psychology, Mayo Clinic, Rochester, Minnesota, USA; Department of Psychiatry and Psychology, Mayo Clinic, Rochester, Minnesota, USA; Department of Psychiatry and Psychology, Mayo Clinic, Rochester, Minnesota, USA; Department of Psychiatry and Psychology, Mayo Clinic, Rochester, Minnesota, USA

**Keywords:** N100, ERP, death/suicide IAT, suicidality

## Abstract

**Background:**

Implicit cognitive markers may assist with the prediction of suicidality beyond clinical risk factors. The aim of this study was to investigate neural correlates associated with the Death/Suicide Implicit Association Test (DS-IAT) via event-related potentials (ERP) in suicidal adolescents.

**Methods:**

Thirty inpatient adolescents with suicidal ideations and behaviors (SIBS) and 30 healthy controls from the community were recruited. All participants underwent 64-channel electroencephalography, DS-IAT, and clinical assessments. Hierarchical generalized linear models with spatiotemporal clustering were used to identify significant ERPs associated with the behavioral outcome of DS-IAT (*D* scores) and group differences.

**Results:**

Behavioral results (*D* scores) showed that the adolescents with SIBS had stronger implicit associations between “death” and “self” than the healthy group (*P *= .02). Within adolescents with SIBS, participants with stronger implicit associations between “death” and “self” reported more difficulty in controllability of suicidal ideation in the past 2 weeks based on the Columbia-Suicide Severity Rating Scale (*P* = .03). For the ERP data, the *D* scores and N100 component over the left parieto-occipital cortex had significant correlations. Significant group differences without behavioral correlation were observed for a second N100 cluster (*P* = .01), P200 (*P* = .02), and late positive potential (5 clusters, all *P *≤ .02). Exploratory predictive models combining both neurophysiological and clinical measures distinguished adolescents with SIBS from healthy adolescents.

**Conclusions:**

Our results suggest that N100 may be a marker of attentional resources involved in the distinction of stimuli that are congruent or incongruent to associations between death and self. Combined clinical and ERP measures may have utility in future refinements of assessment and treatment approaches for adolescents with suicidality.

**Significance Statement**

Combining behavioral outcomes with ERPs may lead to better predictive models for risk assessment in clinical populations, including high-risk groups such as suicidal patients.

## INTRODUCTION

Suicide rates in adolescents have increased at an alarming rate in the past decade despite the knowledge of well-established risk factors, rigorous screening methods, and development of early intervention strategies (Curtin, 2020). A major flaw in current clinical approaches for adolescents with suicidality is the reliance on subjective reports that may be confounded by factors such as fluctuating course of suicidal thoughts and behaviors, implicit factors, lack of insight, intent to avoid involuntary treatment, and fear of stigma (Rickwood et al., 2005; Rowe et al., 2014; Jones et al., 2019; Heinsch et al., 2020). These factors are further complicated in adolescents by the rapid neurodevelopmental changes at the structural and functional levels that influence affect regulation, executive functioning, and impulse control (Cox Lippard et al., 2014; Guyer et al., 2016; Johnston et al., 2017). A greater understanding of the neurophysiologic processes underpinning implicit aspects of suicidality in adolescents would improve assessments of suicidality and guide the development of novel brain-based interventions.

Previous studies have explored neuroimaging correlates of explicit suicidality and the task-based measures of cognitive processes that may be associated with suicidality (Renteria et al., 2017; Schmaal et al., 2020; Auerbach et al., 2021). However, only a few studies have investigated neural correlates of the implicit aspects of suicidality. Most prior studies have used the Implicit Association Test (IAT) to assess implicit suicidality. The IAT, developed by Greenwald et al. (Greenwald et al., 1998), provides an indirect measure of attitudes, stereotypes, and self-concepts. In the past 12 years, the Death/Suicide version of the IAT (DS-IAT) has gained interest. In the DS-IAT, a *D* score is calculated for each individual based on the reaction time differences between the life-congruent and death-congruent blocks, with positive values indicating congruency between “death” and “self” and negative values indicating congruency between “life” and “self.” Several studies have demonstrated correlations between *D* score and past–present–future suicidal behavior (Nock et al., 2010; Randall et al., 2013; Barnes et al., 2017; Tello et al., 2020) in various clinical populations, including adolescents (Glenn et al., 2017; Glenn et al., 2019; Millner et al., 2019). Two prior studies regarding neural correlates of implicit aspects of suicidality were mostly limited to healthy controls, with a few suicidal patients (n = 4) included as proof of concept, and did not demonstrate direct correlations between behavioral measures of the DS-IAT task (*D* score) and imaging findings (Ballard et al., 2019, 2020). Two other studies were limited to structural magnetic resonance imaging findings and did not have control groups (Ho et al., 2018, 2021).

Research regarding event-related potentials (ERPs) may parse subcortical and cortical processes and may provide an opportunity to study perceptual and attentional networks supporting implicit cognition. Previous studies investigating ERP correlates of the behavioral effects of IATs have shown associations of IAT performance with both early and late ERP components. Conflicting results have been most likely related to the great variability in target concepts, measurement techniques, outcome measures, and study populations. Differences in earlier ERP components (such as P100, N100, and N200) have been attributed to selective attention and quicker discrimination/detection of the stimulus (Fleischhauer et al., 2014; Healy et al., 2015; Schiller et al., 2016), whereas differences in later ERP components (P300, N400, and late positive complex/late positive potential [LPP]) have been attributed to resource localization, memory retrieval, decision-making, and semantic processes (Williams and Themanson, 2011; Egenolf et al., 2013) or simply the longer time needed to process the same information for incongruent trials (Schiller et al., 2016). Even though multiple studies have investigated ERP correlates of common IAT tasks, to our knowledge no study has investigated ERPs measured during DS-IATs. Moreover, most of the prior ERP studies in other IATs were in healthy controls, did not account for the spatiotemporal distribution of the effects, and compared congruent trials with incongruent trials without examining the correlation between the intended measure of IAT (*D* score) and ERPs.

We aimed to investigate neural correlates of DS-IAT findings in adolescents with suicidal ideation and behaviors (SIBS) compared with a control group of healthy adolescents. Based on the previous literature, we hypothesized that adolescents with SIBS would have stronger associations between “death” and “self” with the DS-IAT compared with the control group and that the severity of explicit suicidal ideations and behaviors in patients would correlate with the strength of the associations between “death” and “self.” Given the lack of consensus regarding the expected ERP components based on an IAT task, we conducted an exploratory analysis using mass univariate analysis with spatiotemporal clustering to identify ERP components associated with DS-IAT.

## METHODS

### Participants

For this case-control study, we recruited 30 adolescents aged 13 to 18 years with suicidal ideation with or without suicidal behaviors admitted to an inpatient child and adolescent psychiatry unit at Mayo Clinic, Rochester, Minnesota, between January 19, 2019, and June 26, 2020. In addition, 30 healthy adolescents with no prior psychiatric diagnoses were recruited as controls from the local community via flyers, word of mouth, and institutional classified ads. Exclusion criteria included active co-occurring substance use disorder (within the past month, with the exception of caffeine and tobacco); neurologic disorders including seizure disorder (excluding febrile seizures in childhood), anoxia history, and head injuries with loss of consciousness for longer than 5 minutes; any nonremovable hair extensions or hair styling that would impede proper EEG recordings; and pregnancy or suspected pregnancy in female participants assessed with urine pregnancy test. For adolescents with SIBS, additional exclusion criteria were active psychosis or mania, antiepileptic medication use, or chronic benzodiazepine use. For healthy participants, any active or past psychiatric diagnoses were exclusionary. All study procedures were approved by the Mayo Clinic Institutional Review Board. Written informed consent was obtained from each participant and from their legal guardians for participants younger than 18 years. Participants were remunerated for their participation in the study. Demographic characteristics are included in Table 1.

**Table 1.** Demographic Characteristics^a^

Characteristic	Adolescents with SIBS (n = 27)	Controls (n = 30)	*P* value^b^
Age, mo	189.0 (13.4)	185.7 (18.3)	.44
Gender			.054
Female	21 (78)	16 (53)	
Male	6 (22)	14 (47)	
Race			.21
American Indian or Alaska Native	0	0	
Asian	0	1 (3)	
African American	0	0	
Native Hawaiian or Pacific Islander	0	0	
White	25 (93)	29 (97)	
More than one	2 (7)	0	
Ethnicity			.13
Hispanic	2 (7)	0	
Not Hispanic	25 (93)	30 (100)	
Pubertal Development Scale score	3.5 (0.4)	3.2 (0.6)	.07
Handedness			.11
Right	22 (81)	24 (80)	
Left	1 (4)	5 (17)	
Mixed	4 (15)	1 (3)	
UTOX^c^			
THC	3 (11)	1 (3)	
Benzodiazepines	1 (3)	0	
Amphetamines	1 (3)	0	
MOP	0	1 (3)	

Abbreviations: MOP, morphine; SIBS, suicidal ideations and behaviors; THC, **tetrahydrocannabinol**; UTOX, urine toxicology results.

^a^Values are mean (SD) or No. of participants (%).

^b^Comparison with *t* test for continuous variables and χ^2^ test for categorical variables.

^c^Presumptive urine toxicology results (UTOX), no participants met the criteria for substance use disorder. The participant with presumptive amphetamines (also positive for THC) was prescribed stimulants which was held on the day of EEG. One participant with presumptive THC had ibuprofen overdose (assumed false positive), 1 was using CBD oil, and the other 2 admitted to THC use but did not meet criteria for substance use disorder. The patient who tested presumptive positive for benzodiazepine at the time of screening did not receive any benzodiazepine at least 24–48 hours before the EEG. The patients with MOP were presumed false positive.

### Clinical and Behavioral Assessments

All participants were screened with the Mini-International Neuropsychiatric Interview for Children and Adolescents (Sheehan et al., 2010), the Columbia-Suicide Severity Rating Scale (C-SSRS) (Posner et al., 2011), urine drug screening, and a urine pregnancy test (as applicable). Data on handedness (Edinburgh Handedness Inventory), pubertal stage (Pubertal Development Scale and Tanner Staging), psychiatric history, medical history, family history (family psychiatric history), social history (education level, employment, household income, legal history, firearms, religion, relationship status), and medication history were also obtained. The Children’s Depression Rating Scale, revised, was used to assess depressive symptom severity (Poznanski et al., 1996). All clinical assessments were performed by the principal investigator (D.D.C.) and supervised by 3 board-certified child and adolescent psychiatrists (J.S., J.L.V., and P.E.C.).

### EEG Tasks and Recordings

All participants underwent EEG recordings during 3 conditions: (1) resting, (2) visual oddball paradigm task, and (3) DS-IAT ERP task. The EEG was recorded with a vertex-referenced, 64-electrode, high-density, saline-soaked HydroCel Geodesic Sensor net and Net Station 5.4 EEG software (Magstim EGI, Eugene, OR, USA). No additional filters were applied other than the hardware filters. EEG was recorded with a sampling rate of 1000 samples per second. Participants were seated in a chair in front of a computer, 50 cm away from the screen. After verification of proper placement of the EEG net and impedance check (<50 kΩ), participants were given instructions explaining the procedures. All participants were asked to rate their suicidal thoughts, intent, and plan on a visual scale of 0 (none) to 10 (highest) before and after EEG to ensure that suicidal thoughts did not intensify following DS-IAT as participants were exposed the suicide- and death-related words. A post-EEG questionnaire was administered to assess how they felt during recordings (eg, sleepy, daydreaming, anxious, concentration difficulty). The resting EEG was performed first followed by either the DS-IAT or the oddball task in a counterbalanced order, which was randomized among participants. The resting EEG and oddball EEG were collected for other research questions, the results of which are not reported here.

### Death/Suicide Implicit Association Test

The DS-IAT (Nock et al., 2010) task was designed with E-Prime (Psychology Software Tools) and adapted for EEG with longer interstimulus duration (1300-1500 milliseconds with random jitter) and more trials (doubled the number of trials) than the original DS-IAT to avoid overlapping ERP components in later latencies and account for anticipated loss of data due to artifacts during EEG recordings. The supplementary Materials (Supplement 1) show the details of the DS-IAT task.

### Data Analysis and Statistics

#### Demographics and Clinical Data

—Demographic and baseline characteristics were compared between the 2 study groups with independent *t* tests for continuous variables and χ^2^ tests for categorical variables.

#### Behavioral Data (DS-IAT)

—Data were extracted from E-Prime and further analyzed with MATLAB R2020 (MathWorks) and Stata/MP 14.1 (StataCorp LLC). DS-IAT *D* scores were calculated for each participant by using the improved algorithm proposed by Greenwald et al. (Greenwald et al., 2003), with modifications as recommended by Richetin et al. (Richetin et al., 2015), including recoding of extreme latencies <300 milliseconds and >3000 milliseconds and combining practice and test blocks. *D* score is based on reaction time differences; values >0 mean overall faster reaction times in death-congruent blocks, whereas negative values indicate faster reaction times with life-congruent blocks. ANCOVA models were used to control for age and gender to test group differences in *D* scores. We also assessed if subcomponents of the C-SSRS (duration, deterrents, reasons, frequency, controllability) differed among the adolescents with SIBS based on their *D* score (those with positive vs negative *D* scores) after controlling for depression severity.

#### EEG Analysis

—EEG data were exported to EEGLAB (Delorme and Makeig, 2004) and further processed using add-on toolboxes and custom scripts, including ERPLAB (Lopez-Calderon and Luck, 2014), TBT (Ben-Shacher, 2020), and LIMO EEG (Pernet et al., 2011, 2015). The supplementary Materials (Supplement 2) provides further details and references.

Statistical analyses were performed with a mass univariate approach and hierarchical generalized linear models with the LIMO EEG toolbox (Pernet et al., 2011). Group differences and the association between ERPs (dependent variable) and the behavioral outcome of the DS-IAT task (*D* score) were assessed with a robust analysis of covariance model. The dependent variable was the contrast between the ERPs elicited during death-congruent and life-congruent trials. All models included *D* score and age as covariates. Results were corrected for multiple testing by using spatiotemporal clustering (Pernet et al., 2015) within each region. The supplementary Materials provide further details and references (Supplement 3), the rationale and approach for mass univariate models (Supplement 4), and receiver operating characteristic curve (ROC) analysis (Supplement 5). A power analysis based on hypotheses for ERPs associated with the DS-IAT and group differences was calculated with G*Power v 3.119. A sample with 27 participants in each group would provide 80% power to detect a medium effect size of f = 0.5, with α = .05 using an ANCOVA model, with EEG measures as the dependent variables, group as the independent variable, and at least 2 covariates.

## RESULTS

### Demographic and Clinical Characteristics

Three participants from the patient group (adolescents with SIBS) dropped out before completing assessments and were excluded from the analysis. Participant characteristics for the patient (n = 27) and control (n = 30) groups are summarized in Tables 1 and 2.

**Table 2.** Patient Clinical Characteristics

Characteristic	Value^a^ (n = 27)
MINI-KID diagnosis	
MDD	27 (100)
ADHD	4 (15)
GAD	17 (63)
SAD	14 (52)
Phobias	4 (15)
Panic attack	5 (19)
PTSD	4 (15)
SUD (in remission)	1 (4)
EDO	3 (11)
MDD episodes	
Single episode, current	15 (56)
Recurrent, current	11 (41)
Past episode	1 (4)
MDD current episode duration, mo	9 (1-48)
CDRS-R score	56.3 (10.3)
Suicidal behaviors^b^	
Lifetime attempt	19 (70)
Past 2 weeks	
Attempt	13 (48)
Interrupted attempt	13 (48)
Aborted attempt	4 (15)
Preparatory	1 (4)
SIB	13 (48)
Suicidal ideation duration, min/24 h	120 (0-600)
Medications	
SSRI/SNRI	
Fluoxetine	8 (30)
Sertraline	2 (7)
Venlafaxine XR	3 (11)
Citalopram	2 (7)
Escitalopram	5 (19)
Duloxetine	2 (7)
Stimulants	2 (7)
Others	
Mirtazapine	2 (7)
Bupropion	1 (4)
Melatonin	2 (7)
Trazadone	1 (4)
Medication status	
No medications	3 (11)
Recent change (within 6 wk)	21 (78)
Stable regimen	3 (11)

Abbreviations: ADHD, attention deficit/hyperactivity disorder; CDRS-R, Children’s Depression Rating Scale-Revised; EDO, eating disorder; GAD, generalized anxiety disorder; MDD, major depressive disorder; MINI-KID, Mini-International Neuropsychiatric Interview for Children and Adolescents; PTSD, posttraumatic stress disorder; SAD, social anxiety disorder; SIB, self-injurious behavior; SSRI/SNRI, serotonin reuptake inhibitor/serotonin-norepinephrine reuptake inhibitor; SUD, substance use disorder.

^a^Values are No. of participants (%), mean (SD), or median (range).

^b^Assessed with the Columbia-Suicide Severity Rating Scale.

### Behavioral Data

The mean *D* score was negative in both adolescents with SIBS (−0.085; SE, 0.1; 95% CI = −0.28 to 0.12) and healthy controls (−0.35; SE, 0.092; 95% CI = −0.53 to −0.16), which suggests stronger associations between “life” and “self” (vs “death” and “self”) in both groups. However, compared with the healthy group, adolescents with SIBS had stronger implicit associations between “death” and “self” (or less strong associations between “life” and “self,” indicated by a mean *D* score closer to zero) (*P* = .02). Within adolescents with SIBS, those who had stronger implicit associations between “death” and “self” (positive *D* score) scored higher in the controllability of suicidal ideations (higher values indicate more difficulties in controlling thoughts) in the past 2 weeks (based on C-SSRS) than did those with stronger implicit associations between “life” and “self” (mean [SD], 3.82 [0.98] vs 2.88 [1.2]; *P* = .03).

There was no significant change in the severity of suicidal thoughts before and after EEG recordings (*P* > .05).

### Event-Related Potentials

For the ERP data, 3 participants from adolescents with SIBS and 6 healthy controls were excluded from the analysis because of too few available epochs (<10 epochs per condition) after data processing. We did not have a predefined range for ERP components given the spatiotemporal cluster analysis conducted in this study.

**Figure 1.**
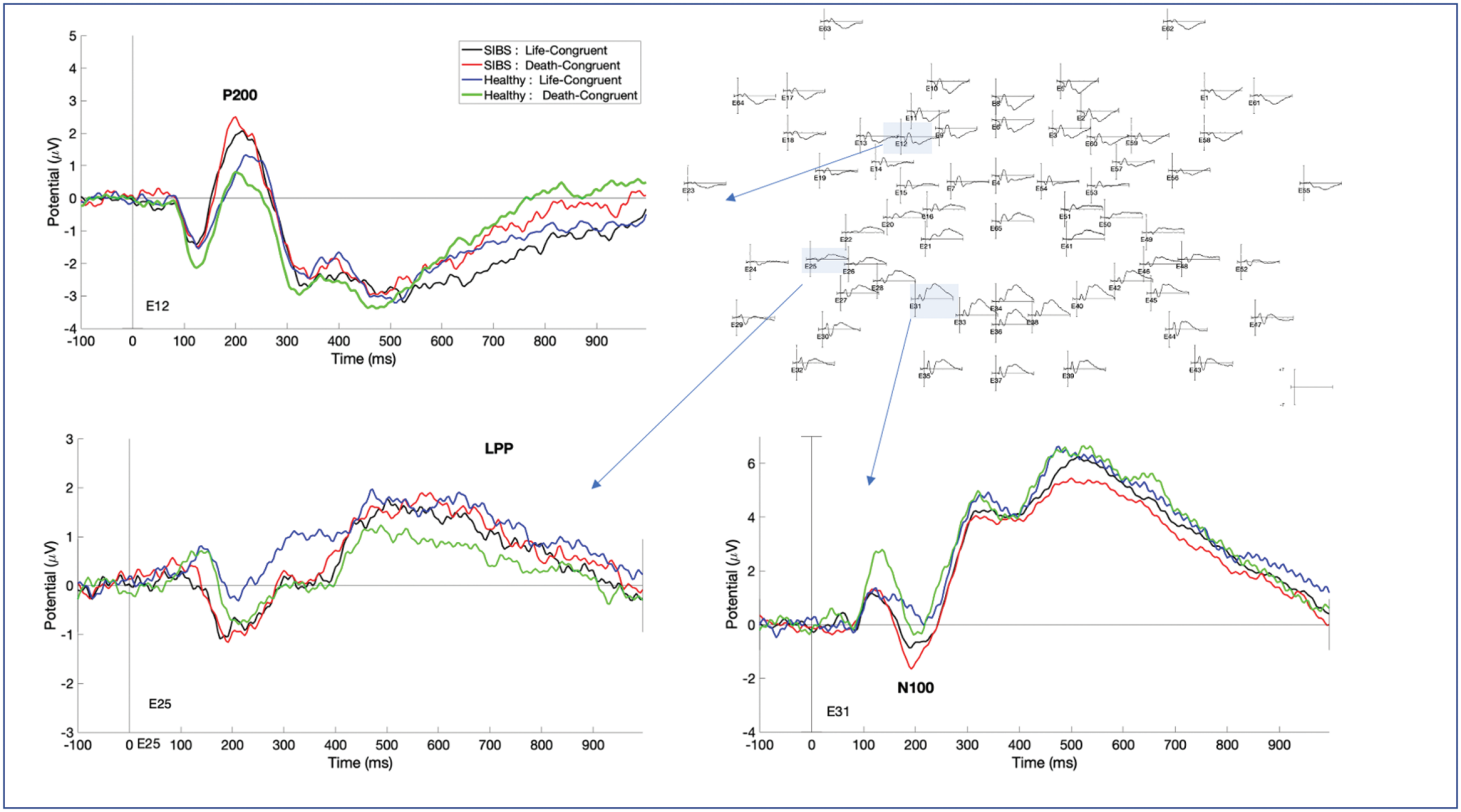
Event-related potentials during the Death/Suicide Implicit Association Test Task. Grand average of for each group and condition showing the distribution of different event-related potentials elicited during the Death/Suicide Implicit Association Test task showing P200, N100, and LPP.

However, visual inspection of the grand average ERPs of all participants (n = 48) was consistent with the expected cortical location of ERPs (Figure 1) in line with previous IAT-ERP research (Fleischhauer et al., 2014; Schiller et al., 2016; Saulnier et al., 2021).

The significant ERP clusters identified after mass univariate analysis are summarized in the supplementary Material (Supplement 6). The reported cluster latencies identify the specific ranges in which statistically significant group difference or the main effect of D score was found within each ERP component.

#### Early ERP Components

##### N100

The visual inspection of the grand averages revealed N100 component over both parietal and occipital areas, between the latencies 160 and 280 ms, following the P100 component (Figure 1). This was consistent with the expected topographic localization of visual N100 but at slightly later latencies than those observed in previous IAT-ERP studies (130–240 milliseconds) (Fleischhauer et al., 2014) and studies investigating visual N100 in other paradigms (Mangun, 1995; Di Russo et al., 2002). There are several factors that can explain the delayed latencies in our study. The IAT task can be considered more difficult than a simple visual detection task requiring a high level of attention to discrimination and categorization of the stimulus. Task difficulty (harder tasks eliciting later N100 components), task type (discrimination vs detection), age, and attention to the stimulus all have been identified as the factors that can alter N100 latency (Mangun and Hillyard, 1991; Mangun, 1995; Di Russo et al., 2002, 2003; Fort et al., 2005; Fleischhauer et al., 2014).

**Figure 2.**
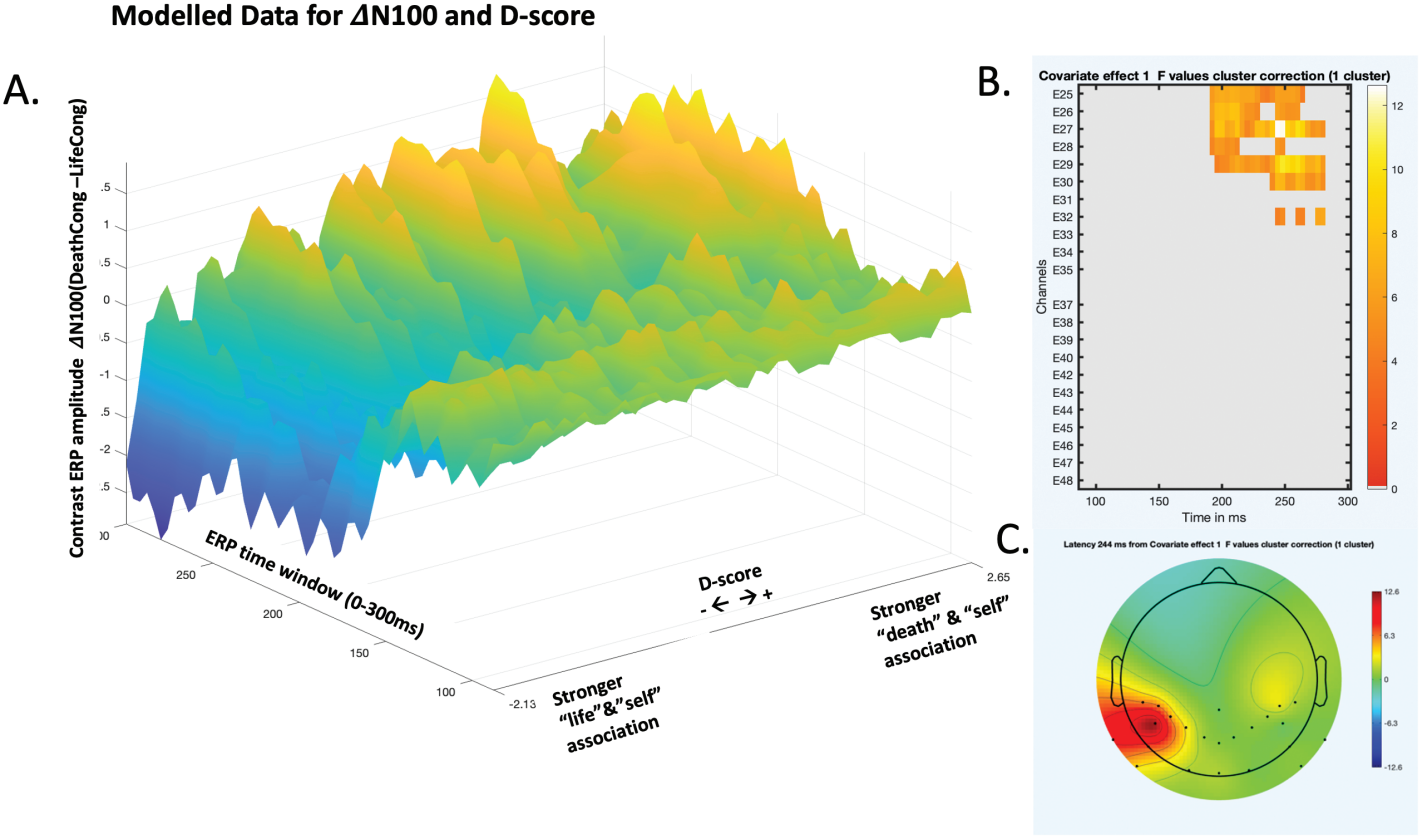
N100 and its association with Death/Suicide Implicit Association Test *D* score. (A) Modelled data with contrast (∆N100) between death-congruent (DeathCong) and life-congruent (LifeCong) trials. A negative contrast means larger (more negative) N100 during death-congruent blocks, whereas a positive contrast means larger N100 during life-congruent blocks. (B) The significant cluster for the observed effect between ∆N100 and *D* score. Color bar represents F values. (C) Topographic distribution of the significant cluster for N100. ERP indicates event-related potential.

A main effect of *D* score was observed over the left parieto-occipital areas with a significant cluster latency starting at 192 milliseconds and ending at 280 milliseconds (cluster *P* = .048). The association between *D* score and the dependent variable (ΔN100, the contrast between N100s elicited during the death-congruent and life-congruent blocks) suggested that participants with more positive *D* scores (those with greater association between “death” and “self”) had more negative N100 amplitudes during the life-congruent blocks than the death-congruent blocks (leading to positive amplitude difference between the 2 conditions). In contrast, participants with more negative *D* scores (greater association between “life” and “self”) had more negative N100 amplitudes during the death-congruent blocks (Figure 2).

**Figure 3.**
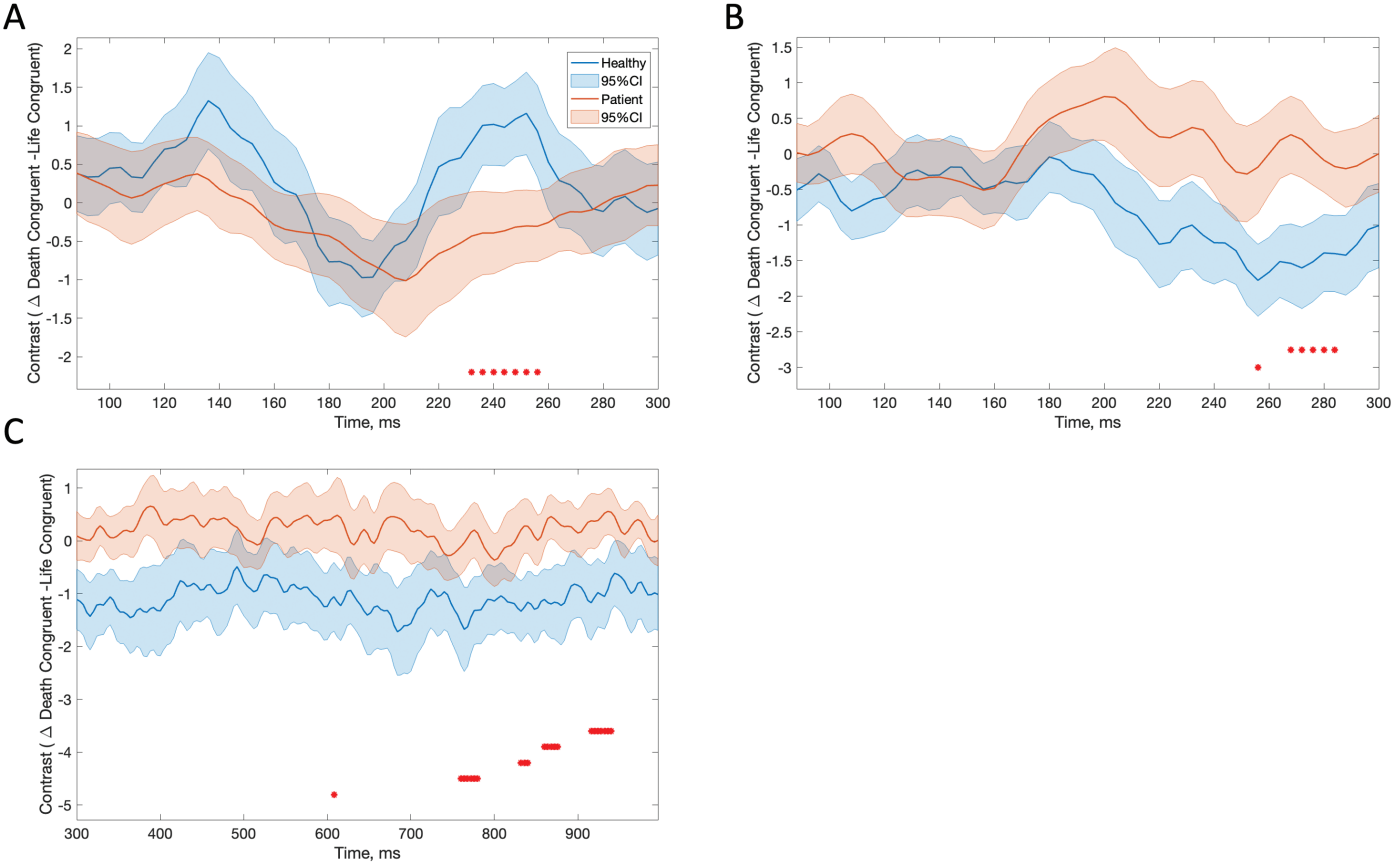
Contrast between death-congruent (Death-Cong) and life-congruent (Life-Cong) data for each group. Graphs show modelled data with 95% CIs. Red asterisks indicate significant latencies for the main effect of group. A, N100, modelled data at channel E31. B, P200, modelled data at channel E12. C, Late positive potential, modelled data at channel E25.

In addition, we observed a main effect of group starting at 236 milliseconds and ending at 256 milliseconds (cluster *P* = .001), also corresponding to later parts of N100. This second N100 cluster was spatially different than the first cluster and included electrodes closer to midline. For this second N100 cluster, adolescents with SIBS had larger N100 during death-congruent blocks than life-congruent blocks, whereas this difference was reversed for healthy controls (Figure 3A).

##### P200

The main effect of group was observed over a cluster corresponding to the left dorsolateral prefrontal cortex area starting at 268 milliseconds and ending at 288 milliseconds (cluster *P* = .002). These latencies correspond to the later parts of P200 as identified through the visual inspection of grand averages for this latency and localization (160–290 milliseconds) (Figure 1). The healthy group had significantly larger P200 during life-congruent blocks than death-congruent blocks, whereas this difference was minimal in adolescents with SIBS (Figure 3B).

#### Late ERP Components

##### LPP

Based on the visual inspection of the grand averages, LPP-like activity was observed over fronto-central and posterior regions (temporo-parietal) starting at around 450 milliseconds and extending at the end of the epoch. We did not observe a main effect of the D score in later ERP components at any latencies. However, a significant main effect of group was found over the fronto-central region (motor cortex, between 868 milliseconds and 876 milliseconds; cluster *P* = .02) and in several clusters in the posterior regions. The posterior regions showed 5 significant clusters, 4 of which were located over the left temporo-parietal areas at latencies varying between 756 and 940 milliseconds. The fifth cluster was over the right temporo-parietal area, although this last cluster had a latency of only 4 milliseconds in these regions and latencies, the healthy group had larger LPP-like activity during life-congruent blocks than death-congruent blocks, whereas adolescents with SIBS had similar or slightly larger LPP-like activity during death-congruent blocks than life-congruent blocks (Figure 3C).

## DISCUSSION

To our knowledge, this was the first study to examine ERP markers of DS-IAT in adolescents with suicidality. The behavioral results of DS-IAT were similar to those of the existing literature, namely that adolescents with SIBS had stronger associations between “death” and “self” than healthy controls that correlated with the controllability of explicit suicidality in the past 2 weeks. Our main ERP results showed that the behavioral outcome of DS-IAT was associated with ΔN100 over the left parieto-occipital cortex such that incongruent stimulus with self-yielded larger N100 components. However, no group differences were found for this specific cluster. In contrast, group differences between adolescents with SIBS and healthy adolescents were observed for a nearby N100 cluster, for P200 over the left prefrontal cortex, and for LPP activity over the temporo-parietal and central areas. However, P200 and LPP activity components and respective regions did not demonstrate correlations with the behavioral outcomes of DS-IAT.

The current findings suggest that N100 is involved in at least 2 separate processes in the DS-IAT task, 1 more relevant to the task itself and the other relevant to group differences. The correlation between parieto-occipital ΔN100 and task-relevant implicit incongruency suggests that the incongruent trials might require more attentional resources for the detection/discrimination of the stimulus. N100 has been widely linked to attentional processing (Haider et al., 1964; Van Voorhis and Hillyard, 1977; Luck et al., 2000; Hopf et al., 2002) and the discrimination of the attended stimuli, with more difficult tasks eliciting larger and more delayed N100 components (Callaway and Halliday, 1982; Vogel and Luck, 2000; Fort et al., 2005). In our study, participants with stronger associations between “death” and “self” (positive *D* score) had larger (more negative) N100 during the life-congruent block, the block that was incongruent to them. This finding may be attributed to higher levels of top-down attentional control required for the difficult trials.

In contrast, significant group differences were observed in a second cluster of N100 that was closer to midline over the parieto-occipital cortex and at the later latencies of N100. Over these regions and latencies, ΔN100 was significantly larger for adolescents with SIBS than the healthy group, which suggests that death-congruent blocks yielded a larger N100 component than the life-congruent blocks in adolescents with SIBS than in the healthy group. Initially this result appears to contradict our main finding of the relationship between ΔN100 and implicit incongruency, because adolescents with SIBS would be expected to have a larger N100 during life-congruent blocks given the inverse relationship between self-congruency and N100 amplitude. However, it is important to note that there were no significant correlations between ΔN100 and behavioral outcomes (*D* scores) over this second cluster unlike the first cluster that showed correlations with *D* scores.

The observed group difference in N100 may be explained by the clinical characteristics of adolescents with SIBS and their impact on attentional processes and N100 rather than solely the behavioral effects of the DS-IAT. Studies investigating ERPs elicited by emotional faces have shown that patients with major depressive disorder (MDD) often exhibit increased N100 amplitude and latency compared with healthy controls in response to “sad” faces (Dai et al., 2016; Hu et al., 2017). However, these findings have not been consistently replicated across the literature. It is possible that bottom-up, stimulus-driven N100 activity may represent attentional bias in adolescents with SIBS during death-congruent trials, but this effect could also be driven by the presence of MDD. Regardless, N100 over this region significantly predicted groups in the exploratory ROC analysis when *D* score was added as a second predictor and therefore may have important implications in distinguishing clinical populations from healthy controls.

The topographic discrepancy between the 2 N100 clusters can be explained by posterior N100 having several subcomponents originating from at least 2 different parts of the cortex: parietal cortex and the lateral occipital cortex (Luck, 2014), with different implications in discrimination and detection of the stimulus (Mangun, 1995; Vogel and Luck, 2000; Di Russo et al., 2002). For example, N100 associated with discrimination has been found to be largest over the occipital cortex and over the left hemisphere (over Brodmann areas 19 and 37) at more delayed latencies (190–220 milliseconds) and has been attributed to top-down modulation of visual stimulus (Hopf et al., 2002).

Group differences were also observed for P200 over the left dorsolateral prefrontal cortex area, along with LPP-like activity over the left parieto-occipital cortex and central areas corresponding to the motor cortex. The healthy group had larger P200 and LPP during life-congruent blocks than death-congruent blocks, whereas the effect was minimal or reversed for adolescents with SIBS. Similar to N100, both P200 and LPP components distinguished groups in the ROC analysis. Group differences over these areas and latencies may be explained by factors such as selective attention (frontal P200), emotional salience, and semantic processing (LPP) independent of the behavioral effect of the DS-IAT because there was no correlation between *D* scores and these ERP components. Both P200 (Hansenne et al., 1996) and LPP elicited during other types of ERP tasks have been implicated in clinical populations with suicidality. For example, LPP has been associated with blunted responses to emotional stimuli (Weinberg et al., 2016; Weinberg et al., 2017), which may also explain the smaller difference elicited between death-congruent and life-congruent conditions for LPP in our results in adolescents with SIBS. Additionally, larger LPP has been associated with compatible trials in other IAT tasks suggesting the role of semantic compatibility and emotional congruence in observed effects (Williams and Themanson, 2011). For adolescents with SIBS, it is possible that death-congruent trials yield similar semantic compatibility and emotional congruence as the life-congruent trials (Williams and Themanson, 2011b).

Similar to N100, P200 has also been associated with attentional processes. Suicide attempters (Allison et al., 2021) and individuals with MDD (Delle-Vigne et al., 2014; Hu et al., 2017) have been shown to have larger P200 amplitudes, indicative of enhanced arousal and attention. In our study, life-congruent trials yielded larger P200 amplitudes than death-congruent trials in healthy controls, whereas this difference was much smaller in adolescents with SIBS. These findings suggest enhanced P200 amplitudes and a possible attentional bias toward death-congruent trials in the SIBS group. Topographically, our P200 results indicate the involvement of the left dorsolateral prefrontal cortex. Visual attention has been associated with left dorsolateral prefrontal cortex activity rather than the right hemisphere, and this may explain why our results were significant only over the left hemisphere (Spooner et al., 2020).

Overall, the correlation between N100 and *D* score can be explained by the early stages of top-down control in the task-related discrimination process, whereas the group differences observed in N100, P200, and LPP may be attributed to both top-down and bottom-up attentional deficits/bias and emotional valence/salience of the trials. Attention deficits and attentional bias have been implicated in numerous studies and remain as a critical area of research in understanding mood disorders and suicide (Becker et al., 1999; Keilp et al., 2008; Keilp et al., 2013; Richard-Devantoy et al., 2016).

There are several limitations of this study. The 2 study groups were recruited from different settings, inpatient vs nonclinical setting, which can limit the generalizability of our results. Our sample size was relatively limited and therefore did not allow testing for additional confounding factors such as the presence of anxiety disorders, attention-deficit/hyperactivity disorder (ADHD), or medication use. For example, ERP studies in ADHD have demonstrated abnormalities in ERP components such as CNV, P200, and P300, attributed to ineffective cognitive modulation (Kaiser et al., 2020). However, in our analysis, only 3 out of 24 patients with SIBS met the criteria for ADHD, indicating a minimal overall impact from these participants. Similarly, both depression and anxiety can contribute to ERP abnormalities, affecting early components (P100, N100) as well as late components (P300), as previously explained above. In our findings, the group differences observed in N100, P200, and LPP-like activity cannot be solely attributed to the presence of SIBS, as all adolescents with SIBS also met criteria for MDD and approximately 63% met criteria for Generalized Anxiety Disorder. It is also worth noting that medication status is another factor that can influence EEG activity and ERP components. For example, studies have shown the effects of SSRIs on β power density (Siepmann et al., 2003) and P300 (Hansenne et al., 1998) in both healthy adults and clinical populations with schizophrenia (Hyun et al., 2011), although results have been inconsistent across studies (Oranje et al., 2008). Therefore, predicting the specific impact of medication on our results would be challenging. Stimulants can also affect ERPs, but they were held on the day of EEG and no patient was taking benzodiazepines or mood stabilizers at the time of EEG because this was an exclusion criteria. It is also important to note that the majority (78%) of the adolescents with SIBS underwent medication changes within the past 6 weeks of study enrollment, which could have affected our results. Another limitation is that even though we showed correlations between both explicit and implicit measures, this does not completely determine whether significant correlations between *D* scores and N100 are indeed specific to a suicide-related IAT or can be observed in other self-concept IATs.

This study showed correlations between behavioral outcomes of the DS-IAT and both ERPs and explicit suicide measures in inpatients adolescents with SIBS. To our knowledge, this is the first study investigating correlations between implicit measures of suicidality (DS-IAT) and event-related potentials in inpatient adolescents compared with healthy controls. Research focusing on inpatient adolescents with recent suicidality is especially crucial because this type of clinical population remains understudied due to the exclusion of participant with severe suicidality requiring hospitalization. Combining behavioral outcomes with ERPs may lead to better predictive models because single measures fail to predict the risk in clinical populations including high-risk groups such as suicidal patients. The future integration and scaling of ERPs measured with EEG for clinical practice may provide opportunities for improved diagnostic and treatment approaches for adolescents with suicidal ideations and behaviors.

## Supplementary Material

pyad039_suppl_Supplementary_Material

## Interest Statement

Dr Croarkin has received research grant support from Mayo Foundation for Medical Education and Research, Neuronetics, Inc.; NeoSync, Inc; and Pfizer, Inc. He has received grant-in-kind (equipment support for investigator-initiated research studies) from Assurex; MagVenture, Inc; and Neuronetics, Inc. He has served on advisory boards for Engrail Therapeutics, Myriad Neuroscience, Procter & Gamble, and Sunovion. Dr Vande Voort has received grant-in-kind (equipment support for investigator-initiated research studies) from Assurex. Dr Lewis receives research grant support from the Brain & Behavior Research Foundation (Alan G. Ross Memorial Investigator), the Klingenstein Third Generation Foundation, and American Foundation for Suicide Prevention; and he previously has served as a site investigator for studies funded by Neuronetics, Inc. and NeoSync, Inc. The other authors have no disclosures or potential conflicts of interest.

## Data Access and Responsibility

Deniz Doruk Camsari, MD, and Paul E. Croarkin, DO, had full access to all the data in the study and take responsibility for the integrity of the data and the accuracy of the data analysis.
