# Supplementary material for: Event-Related Potential Markers of Suicidality in Adolescents
Source: Int J Neuropsychopharmacol. 2023 Jul 9;26(8):566–75. doi: 10.1093/ijnp/pyad039 (PMC10464930; doi:10.1093/ijnp/pyad039)
Supplement: pyad039_suppl_Supplementary_Material [file pyad039_suppl_supplementary_material.docx]

**Supplementary Materials**

**Supplement 1. DS-IAT Task**

The task consisted of 7 blocks. See table 1 for the distribution of trials. The positions of blocks 1, 3, and 4 are switched with those of 5, 6, and 7 in half of the participants, similar to the original IAT design. In each trial a word is presented as the ‘stimulus’ (font: Arial, size: 18) and appeared in the middle of the screen. Participants were asked to respond by hitting the ‘E’ key if the stimulus belonged to the category name indicated on the left upper corner of the screen and the ‘I’ key if the stimulus belonged to the category name indicated on the right upper corner. In Blocks 3, 4, 6, and 7 words belonging to the Death or Life categories [thrive, survive, alive, breathing, live, die, deceased, funeral, suicide, lifeless] alternated with words belonging to Me or Not Me [myself, I, self, mine, my, they, their, other, them, theirs] categories. The number of trials and interstimulus interval have been adapted for an ERP study. Figure 1 shows examples for Death-Congruent and Life-Congruent trials.

| Block no. | # Trials | Left side category name | Right side category name | Block condition |
| --- | --- | --- | --- | --- |
| 1 | 20 | Death | Life |  |
| 2 | 20 | Me | Not Me |  |
| 3 | 40 | Death/Me | Life/Not Me | Death-Congruent |
| 4 | 80 | Death/Me | Life/Not Me | Death-Congruent |
| 5 | 20 | Life | Death |  |
| 6 | 40 | Life/Me | Death/Not Me | Life-Congruent |
| 7 | 80 | Life/Me | Death/Not Me | Life-Congruent |

Table 1. DS-IAT Trials. Distribution of trials for each block


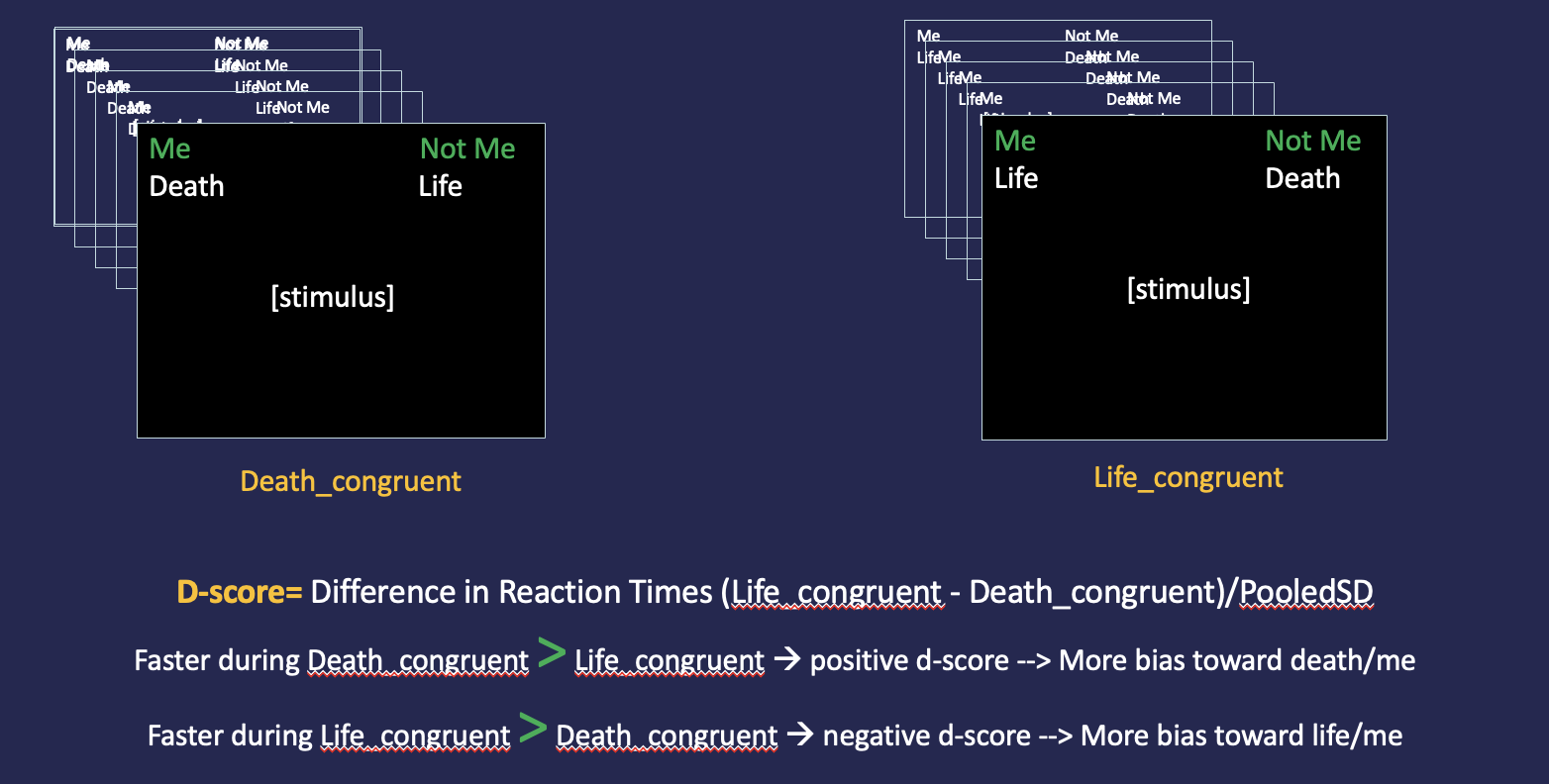


Figure 1. Examples for Death-Congruent and Life-Congruent trials. *D* score was calculated based on the difference in reaction times in each block.

**Supplement 2.** Distribution of EEG channels over the scalp. Purple electrodes represent posterior electrodes and blue electrodes represent frontocentral electrodes. EEGLAB STUDY structure was used to extract and process single-trial measures from all participants automatically.


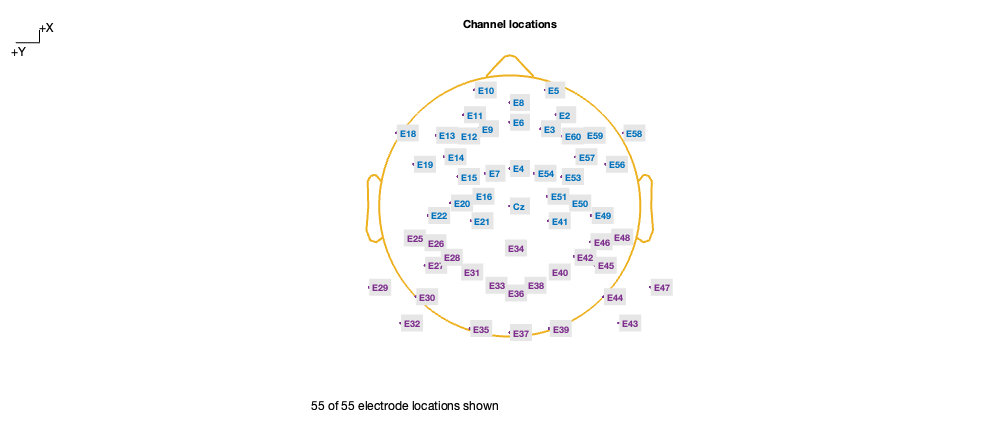


**Supplement 3. EEG Data Processing**

EEG data were exported to EEGlab and further analyzed using add-on toolboxes and scripts. Data were filtered (0.1-40 Hz bandpass), epoched (from –100 ms to +1,000 ms) and baseline corrected (–100 ms to 0 ms).

Automatic artifact detection was performed using a moving window peak-to-peak artifact threshold (-50 mV to +50 mv) tool via ERPlab. Epochs including eye blinks and large artifacts detected at this step were automatically discarded. We chose to discard epochs with eye blinks instead of other available correction methods (such as with ICA), as removing the blink component from epochs may not be sufficient to address the neural effects of blinks on neural processes (1, 2).A second automatic artifact detection algorithm was run using EEGLAB's TBT plugin (3). Within each epoch, channels that exceeded a differential average amplitude of 50 μV were marked for correction and then subsequently interpolated within the epoch. Channels that were marked as bad on more than 30% of all epochs were interpolated as a whole channel. Epochs having more than 30 bad channels were excluded. Each EEG recording was manually inspected before and after each automated artifact detection/rejection process. Data were re-reference to average of all channels and downsampled to 250 Hz. Following data cleaning, the mean (±SD) number of trials included in the analysis was 55 (±27) for death-congruent trials and 51 (±32) for life-congruent trials, out of an initial total of 120 trials for each trial type. This represents 42-45% of the original trials, all of which were included in the statistical analysis.

**Supplement 4. EEG Statistical Analysis**

EEGLAB STUDY structure was used to extract and process single-trial measures from all participants automatically. Statistical analyses were performed using a mass univariate approach and hierarchical general linear models using the LIMO EEG toolbox (4). At the first level of the analysis, within-participant general linear models were used to analyze the model parameters (beta parameters) using trial-by-trial data for each participant and block condition (Death-Congruent and Life-Congruent) at each time point and at each channel independently. Parameter estimates were obtained using Ordinary Least Squares. Contrast between the 2 conditions (Death-Congruent> Life-Congruent) were computed for each participant. Since *D* score is based on the reaction time differences between Death-Congruent and Life-Congruent blocks, we calculated the contrasts between the two conditions that would allow us to examine the association between *D* score and ERP differences more accurately.

At the second level (between-participant) of the analysis, a robust ANCOVA model was used to analyze the group differences and the relationship between the contrasts and *D* scores controlled by age (contrast as the dependent variable, group as the independent variable, *D* score and age as covariates). Results are reported corrected for multiple testing using spatiotemporal clustering (5).

Mass univariate models have greater power than other traditional averaging approaches when a priori time windows and spatial regions are identified. Based on the previous IAT-ERP research, expected ERP components were P100, N100, P200, P300, MFN, N400, and LPP. Given known locations and expected time ranges of these ERP components, we divided our analysis in 2 larger regions of interest (ROI), frontocentral (32 electrodes, ROI for anterior N100, P200, MFN/N400) and posterior including parietal and occipital areas (23 electrodes, ROI for P100, posterior N100, P300/LPP).

Periauricular and facial electrodes, even though included in average referencing, were excluded from the ERP analysis. Earlier components (P100, N100, P200) and later components (P300, LPP, N400) were analyzed separately.

**Supplement 5**. **ROC Analysis**

We ran separate exploratory analysis based on the main ERP results using logistic regression models to test whether ERPs can distinguish between the groups. For the purposes of this analysis, we considered group as the dependent variable and the ERP data (the contrast between the death-congruent and life-congruent trials) as the main predictor controlled with *D* score and age. For each ERP component, the channels and the latency with the maximum F values within each significant cluster were chosen. These included channel E12 at 268 ms (corresponds to P200 over DLPFC) and E31 at 248 ms (corresponds to N100 over left parieto-occipital region) for earlier components (<300 ms). For later components (LPP-like activity) it included the electrodes E20 at 868 ms (over C3/left, motor cortex) and E25 at 868 ms (left temporoparietal area). Receiver operating characteristic (ROC) analysis was performed to assess the sensitivity and specify of ERPs in distinguishing groups. E46 was not tested because the cluster was significant for less than 5 ms.

For early components 1) ΔN100 significantly predicted group (*P*=.008, B=0.661) with an area under the curve (AUC) of 0.8095; 2) ΔP200 over the left DLPFC significantly predicted group (*P*=.008, B= –0.599) with an AUC of 0.7865; and for later components 3) ΔLPP-like activity over both the left motor cortex (*P*=.01, AUC=0.7778) and left temporoparietal area (*P*=.006, AUC=0.7986) significantly predicted group.

**Supplement 6. Significant Clusters**

|  | **# Significant clusters** | **Cluster *P* value/Max F value** | **Electrodes within clusters** | **Latency (range)**  **for maximum F value** | **ERP** |
| --- | --- | --- | --- | --- | --- |
| **Early Components [90-300 ms]** |  |  |  |  |  |
| **Fronto-Central**  Main Effect *D* Score  Main Effect Group  Age | NS  Cluster 1  NS | .002/9.75438 | E6,E7, E9,**E12**,E14, E15,E16, E20 | **268** (268-288) | P200 |
| **Posterior**  Main Effect *D* Score  Main Effect Group  Age | Cluster 1  Cluster 1  NS | .048/12.6434  .001/10.7435 | E25, E26, **E27**, E28, E29, E30,E32  **E31**, E33,E34,E36,E37 | 244 (192-280)  **248** (236-256) | N100  N100 |
| **Late Components [300-1,000 ms]** |  |  |  |  |  |
| **Fronto-Central**  Main Effect *D* Score  Main Effect Group  Age | NS  Cluster 1  NS | .02/7.40831 | E14, E15, E19, **E20**, E22 | 868 (868-876) | LPP |
| **Posterior**  Main Effect *D* Score  Main Effect Group  Age | NS  Cluster 1  Cluster 2  Cluster 3  Cluster 4  Cluster 5  NS | .005/7.42099  .02/8.6443  .008/10.2168  .005/8.48883  .02/7.59831 | **E25**, E26, E27, E28, E30  **E25**, E26, E27, E28, E30  **E25**, E26, E27, E28, E30  **E25**, E26, E27, E28, E30  E42, E43, E45, **E46,** E48 | 776 (756-780)  836 (832-840)  **868** (860-876)  932 (916-940)  **872** (872-876) | LPP |

1. Bonfiglio L, Sello S, Andre P, Carboncini MC, Arrighi P, Rossi B. Blink-related delta oscillations in the resting-state EEG: a wavelet analysis. Neuroscience letters. 2009;449(1):57-60.

2. Bonfiglio L, Sello S, Carboncini MC, Arrighi P, Andre P, Rossi B. Reciprocal dynamics of EEG alpha and delta oscillations during spontaneous blinking at rest: a survey on a default mode-based visuo-spatial awareness. International Journal of Psychophysiology. 2011;80(1):44-53.

3. Ben-Shachar MS. TBT: Reject and Interpolate channels on a trial-by-trial basis ((Version v2.6.0) EEGLAB plugin).2020.

4. Pernet CR, Chauveau N, Gaspar C, Rousselet GA. LIMO EEG: a toolbox for hierarchical LInear MOdeling of ElectroEncephaloGraphic data. Computational intelligence and neuroscience. 2011;2011.

5. Pernet CR, Latinus M, Nichols T, Rousselet G. Cluster-based computational methods for mass univariate analyses of event-related brain potentials/fields: A simulation study. Journal of neuroscience methods. 2015;250:85-93.
